# 2,2′-Diisopropoxy-5,5′-methyl­enedi­benz­alde­hyde

**DOI:** 10.1107/S1600536812028048

**Published:** 2012-06-23

**Authors:** G. Suresh, V. Sabari, A. Devaraj, M. Bakthadoss, S. Aravindhan

**Affiliations:** a Department of Physics, Presidency College (Autonomous), Chennai 600 005, India; b Department of Organic Chemistry, University of Madras, Chennai 600 025, India

## Abstract

Mol­ecules of the title compound, C_21_H_24_O_4_, are located on a twofold rotation axis running through the central methyl­ene C atom. The aldehyde group is coplanar with the benzene ring [C—C—C—O = 175.7 (4) °].

## Related literature

For related salicyl­aldehyde compounds, see: Qiu *et al.* (2009 ▶); Yu *et al.* (2007 ▶); Wang *et al.* (2009 ▶).
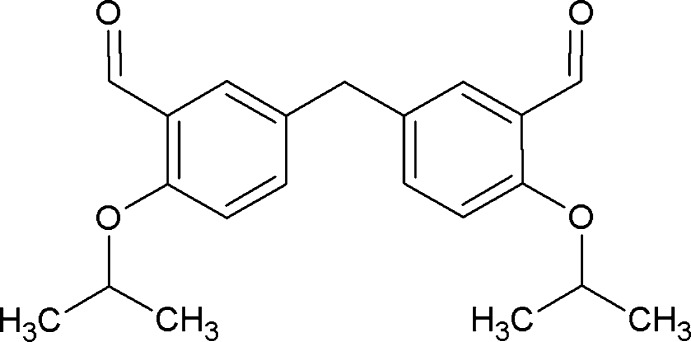


## Experimental

### 

#### Crystal data

C_21_H_24_O_4_
*M* *_r_* = 340.40
Orthorhombic, 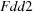

*a* = 26.337 (9) Å
*b* = 28.349 (10) Å
*c* = 4.990 (2) Å
*V* = 3726 (2) Å^3^
*Z* = 8
Mo *K*α radiation
μ = 0.08 mm^−1^
*T* = 293 K
0.35 × 0.25 × 0.20 mm

#### Data collection

Bruker SMART APEXII area-detector diffractometer
Absorption correction: multi-scan (*SADABS*; Bruker, 2008 ▶) *T*_min_ = 0.972, *T*_max_ = 0.984
4329 measured reflections
1022 independent reflections
886 reflections with *I* > 2σ(*I*)
*R*_int_ = 0.035

#### Refinement

*R*[*F* ^2^ > 2σ(*F* ^2^)] = 0.040
*wR*(*F* ^2^) = 0.114
*S* = 1.06
1022 reflections
114 parameters
1 restraint
H-atom parameters constrained
Δρ_max_ = 0.17 e Å^−3^
Δρ_min_ = −0.13 e Å^−3^

### 

Data collection: *APEX2* (Bruker, 2008 ▶); cell refinement: *SAINT* (Bruker, 2008 ▶); data reduction: *SAINT*; program(s) used to solve structure: *SHELXS97* (Sheldrick, 2008 ▶); program(s) used to refine structure: *SHELXL97* (Sheldrick, 2008 ▶); molecular graphics: *ORTEP-3* (Farrugia, 1997 ▶); software used to prepare material for publication: *SHELXL97* and *PLATON* (Spek, 2009 ▶).

## Supplementary Material

Crystal structure: contains datablock(s) I, global. DOI: 10.1107/S1600536812028048/bt5945sup1.cif

Structure factors: contains datablock(s) I. DOI: 10.1107/S1600536812028048/bt5945Isup2.hkl

Supplementary material file. DOI: 10.1107/S1600536812028048/bt5945Isup3.cml

Additional supplementary materials:  crystallographic information; 3D view; checkCIF report

## Figures and Tables

**Fig. 1.**
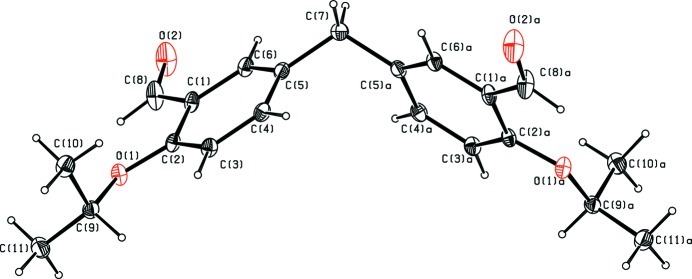
The molecular structure of the title compound, with displacement ellipsoids drawn at the 10% probability level.

**Fig. 2.**
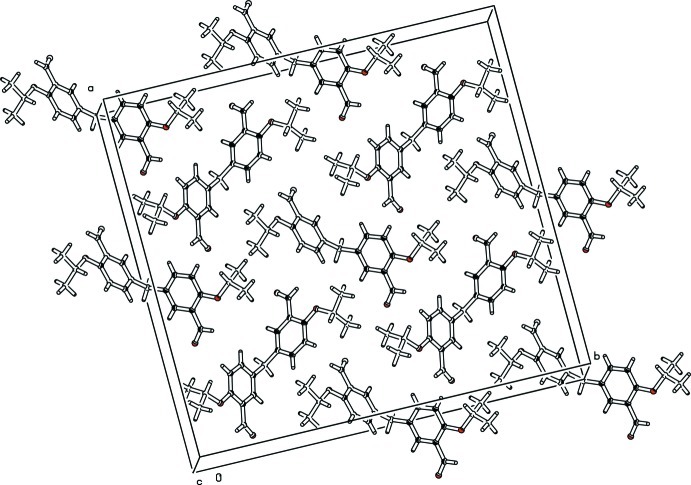
A view of the crystal packing.

## supplementary crystallographic information

### Comment

Salicylaldehyde and its derivatives are widely used in the construction of metal complexes (Qiu *et al.*, 2009; Wang *et al.*,2009; Yu *et al.*,2007).

X-Ray analysis confirms the molecular structure and atom connectivity as illustrated in (Fig. 1). There os one half molecule in the asymmetric unit located on a two-fold rotation axis. The aldehyde group is coplanar with the benzene ring [C2—C1—C8—O2 = 175.7 (4) °].

The crystal packing is stabilized by weak C-H···O hydrogen bonds.

### Experimental

To a stirred solution of 5,5'-methylenebis(2-hydroxybenzaldehyde) (0.50 g, 1.95 mmol) in CH_3_CN (20 ml) was added K_2_CO_3_ (1.35 g, 9.76 mmol) and stirred for 15 min. To this, isopropylbromide (0.40 ml, 4.29 mmol) was added and stirred for 24 h at rt. After completion of the reaction as indicated by TLC, the reaction mixture was concentrated and the resulting crude mass was diluted with water (15 ml) and extracted with ethyl acetate (3 × 15 ml). The combined organic layer was washed with brine (2 × 10 ml) and dried over anhydrous Na_2_SO_4_.The organic layer was concentrated and the solid thus obtained was washed with ethylacetate-hexanes (1:9) to afford the compound 5,5'-Methylenebis(2-isopropoxybenzaldehyde) as a colourless solid in 95% yield.

### Refinement

Hydrogen atom were found in a difference electron density map and subsequently treated as riding atoms with distances of 0.96 Å (CH_3_), 0.97 Å (CH_2_), 0.98 Å (tertiary CH) or 0.93 Å (aromatic CH). *U*_iso_(H) was set to 1.2*U*_eq_(C) or 1.5*U*_eq_(C_methyl_), respectively. Due to the absence of anomalous scatterers, Friedel pairs were merged.

### Crystal data

**Table:**

C_21_H_24_O_4_	*F*(000) = 1456
*M**_r_* = 340.40	*D*_x_ = 1.214 Mg m^−^^3^
Orthorhombic, *F**d**d*2	Mo *K*α radiation, λ = 0.71073 Å
Hall symbol: F 2 -2d	Cell parameters from 1022 reflections
*a* = 26.337 (9) Å	θ = 3.1–26.0°
*b* = 28.349 (10) Å	µ = 0.08 mm^−^^1^
*c* = 4.990 (2) Å	*T* = 293 K
*V* = 3726 (2) Å^3^	Orthorhombic, colourless
*Z* = 8	0.35 × 0.25 × 0.20 mm

### Data collection

**Table:**

Bruker SMART APEXII area-detector diffractometer	1022 independent reflections
Radiation source: fine-focus sealed tube	886 reflections with *I* > 2σ(*I*)
Graphite monochromator	*R*_int_ = 0.035
ω and φ scans	θ_max_ = 26.0°, θ_min_ = 3.1°
Absorption correction: multi-scan (*SADABS*; Bruker, 2008)	*h* = −32→32
*T*_min_ = 0.972, *T*_max_ = 0.984	*k* = −31→34
4329 measured reflections	*l* = −6→4

### Refinement

**Table:**

Refinement on *F*^2^	Primary atom site location: structure-invariant direct methods
Least-squares matrix: full	Secondary atom site location: difference Fourier map
*R*[*F*^2^ > 2σ(*F*^2^)] = 0.040	Hydrogen site location: inferred from neighbouring sites
*wR*(*F*^2^) = 0.114	H-atom parameters constrained
*S* = 1.06	*w* = 1/[σ^2^(*F*_o_^2^) + (0.0561*P*)^2^ + 2.1412*P*] where *P* = (*F*_o_^2^ + 2*F*_c_^2^)/3
1022 reflections	(Δ/σ)_max_ < 0.001
114 parameters	Δρ_max_ = 0.17 e Å^−^^3^
1 restraint	Δρ_min_ = −0.13 e Å^−^^3^

### Special details

**Table:**

Geometry. All e.s.d.'s (except the e.s.d. in the dihedral angle between two l.s. planes) are estimated using the full covariance matrix. The cell e.s.d.'s are taken into account individually in the estimation of e.s.d.'s in distances, angles and torsion angles; correlations between e.s.d.'s in cell parameters are only used when they are defined by crystal symmetry. An approximate (isotropic) treatment of cell e.s.d.'s is used for estimating e.s.d.'s involving l.s. planes.
Refinement. Refinement of *F*^2^ against ALL reflections. The weighted *R*-factor *wR* and goodness of fit *S* are based on *F*^2^, conventional *R*-factors *R* are based on *F*, with *F* set to zero for negative *F*^2^. The threshold expression of *F*^2^ > σ(*F*^2^) is used only for calculating *R*-factors(gt) *etc*. and is not relevant to the choice of reflections for refinement. *R*-factors based on *F*^2^ are statistically about twice as large as those based on *F*, and *R*- factors based on ALL data will be even larger.

### Fractional atomic coordinates and isotropic or equivalent isotropic displacement parameters (Å^2^)

**Table:**

	*x*	*y*	*z*	*U*_iso_*/*U*_eq_	Occ. (<1)
O1	0.15629 (6)	0.10388 (6)	0.7445 (5)	0.0632 (5)	
C4	0.25293 (9)	0.18516 (9)	0.9981 (6)	0.0580 (6)	
H4	0.2873	0.1919	0.9767	0.070*	
C2	0.18085 (8)	0.13881 (8)	0.8776 (6)	0.0499 (6)	
C7	0.2500	0.2500	1.3451 (9)	0.0711 (11)	
H7A	0.2245	0.2642	1.4598	0.085*	0.50
H7B	0.2755	0.2358	1.4598	0.085*	0.50
C1	0.15181 (8)	0.16511 (8)	1.0577 (6)	0.0552 (7)	
C3	0.23209 (8)	0.14955 (8)	0.8487 (6)	0.0572 (7)	
H3	0.2521	0.1326	0.7288	0.069*	
C5	0.22521 (9)	0.21160 (8)	1.1795 (5)	0.0546 (6)	
C6	0.17461 (9)	0.20091 (8)	1.2046 (6)	0.0578 (7)	
H6	0.1549	0.2182	1.3240	0.069*	
C9	0.18476 (10)	0.06998 (9)	0.5895 (6)	0.0621 (7)	
H9	0.2076	0.0865	0.4661	0.075*	
O2	0.07084 (7)	0.17443 (8)	1.2544 (9)	0.1226 (13)	
C10	0.21526 (11)	0.03853 (10)	0.7716 (8)	0.0760 (9)	
H10A	0.2397	0.0571	0.8674	0.114*	
H10B	0.2325	0.0151	0.6667	0.114*	
H10C	0.1930	0.0233	0.8968	0.114*	
C11	0.14556 (13)	0.04302 (12)	0.4324 (8)	0.0882 (11)	
H11A	0.1274	0.0643	0.3176	0.132*	
H11B	0.1222	0.0281	0.5537	0.132*	
H11C	0.1620	0.0194	0.3255	0.132*	
C8	0.09748 (9)	0.15523 (10)	1.0940 (10)	0.0888 (13)	
H8	0.0828	0.1325	0.9845	0.107*	

### Atomic displacement parameters (Å^2^)

**Table:**

	*U*^11^	*U*^22^	*U*^33^	*U*^12^	*U*^13^	*U*^23^
O1	0.0503 (9)	0.0492 (9)	0.0902 (13)	−0.0024 (7)	0.0055 (10)	−0.0102 (10)
C4	0.0436 (11)	0.0618 (14)	0.0686 (15)	−0.0084 (10)	0.0086 (12)	0.0086 (14)
C2	0.0445 (11)	0.0388 (11)	0.0663 (15)	0.0025 (9)	0.0052 (12)	0.0066 (11)
C7	0.079 (2)	0.074 (3)	0.060 (2)	−0.0213 (19)	0.000	0.000
C1	0.0430 (11)	0.0383 (11)	0.0844 (19)	0.0028 (9)	0.0120 (13)	0.0036 (13)
C3	0.0477 (12)	0.0544 (14)	0.0695 (16)	−0.0004 (10)	0.0158 (12)	−0.0006 (14)
C5	0.0576 (12)	0.0495 (13)	0.0568 (15)	−0.0065 (10)	0.0027 (12)	0.0081 (12)
C6	0.0560 (12)	0.0444 (12)	0.0730 (18)	0.0018 (10)	0.0181 (13)	0.0005 (14)
C9	0.0704 (15)	0.0549 (14)	0.0609 (15)	−0.0082 (12)	0.0218 (14)	−0.0046 (13)
O2	0.0639 (12)	0.0834 (15)	0.221 (4)	−0.0002 (10)	0.0623 (19)	−0.031 (2)
C10	0.0793 (17)	0.0574 (15)	0.091 (2)	0.0121 (13)	0.0235 (18)	−0.0004 (17)
C11	0.098 (2)	0.082 (2)	0.084 (2)	−0.0235 (17)	0.011 (2)	−0.016 (2)
C8	0.0464 (13)	0.0554 (15)	0.164 (4)	0.0008 (12)	0.024 (2)	−0.018 (2)

### Geometric parameters (Å, º)

**Table:**

O1—C2	1.357 (3)	C5—C6	1.372 (3)
O1—C9	1.444 (3)	C6—H6	0.9300
C4—C3	1.370 (4)	C9—C10	1.505 (4)
C4—C5	1.384 (4)	C9—C11	1.505 (4)
C4—H4	0.9300	C9—H9	0.9800
C2—C3	1.391 (3)	O2—C8	1.196 (5)
C2—C1	1.396 (3)	C10—H10A	0.9600
C7—C5^i^	1.514 (4)	C10—H10B	0.9600
C7—C5	1.515 (4)	C10—H10C	0.9600
C7—H7A	0.9700	C11—H11A	0.9600
C7—H7B	0.9700	C11—H11B	0.9600
C1—C6	1.389 (3)	C11—H11C	0.9600
C1—C8	1.469 (3)	C8—H8	0.9300
C3—H3	0.9300		
			
C2—O1—C9	120.03 (18)	C5—C6—H6	118.9
C3—C4—C5	122.9 (2)	C1—C6—H6	118.9
C3—C4—H4	118.5	O1—C9—C10	110.4 (3)
C5—C4—H4	118.5	O1—C9—C11	105.1 (2)
O1—C2—C3	124.9 (2)	C10—C9—C11	112.3 (2)
O1—C2—C1	116.35 (18)	O1—C9—H9	109.6
C3—C2—C1	118.8 (2)	C10—C9—H9	109.6
C5^i^—C7—C5	113.9 (4)	C11—C9—H9	109.6
C5^i^—C7—H7A	108.8	C9—C10—H10A	109.5
C5—C7—H7A	108.8	C9—C10—H10B	109.5
C5^i^—C7—H7B	108.8	H10A—C10—H10B	109.5
C5—C7—H7B	108.8	C9—C10—H10C	109.5
H7A—C7—H7B	107.7	H10A—C10—H10C	109.5
C6—C1—C2	119.6 (2)	H10B—C10—H10C	109.5
C6—C1—C8	119.7 (3)	C9—C11—H11A	109.5
C2—C1—C8	120.8 (2)	C9—C11—H11B	109.5
C4—C3—C2	119.6 (2)	H11A—C11—H11B	109.5
C4—C3—H3	120.2	C9—C11—H11C	109.5
C2—C3—H3	120.2	H11A—C11—H11C	109.5
C6—C5—C4	116.9 (2)	H11B—C11—H11C	109.5
C6—C5—C7	121.8 (2)	O2—C8—C1	124.6 (3)
C4—C5—C7	121.2 (2)	O2—C8—H8	117.7
C5—C6—C1	122.2 (2)	C1—C8—H8	117.7
			
C9—O1—C2—C3	9.8 (4)	C5^i^—C7—C5—C6	−122.2 (3)
C9—O1—C2—C1	−169.6 (2)	C5^i^—C7—C5—C4	58.8 (2)
O1—C2—C1—C6	178.9 (2)	C4—C5—C6—C1	0.4 (4)
C3—C2—C1—C6	−0.5 (4)	C7—C5—C6—C1	−178.6 (3)
O1—C2—C1—C8	−0.9 (4)	C2—C1—C6—C5	0.0 (4)
C3—C2—C1—C8	179.7 (3)	C8—C1—C6—C5	179.8 (3)
C5—C4—C3—C2	−0.1 (4)	C2—O1—C9—C10	68.9 (3)
O1—C2—C3—C4	−178.8 (2)	C2—O1—C9—C11	−169.7 (2)
C1—C2—C3—C4	0.6 (4)	C6—C1—C8—O2	−4.1 (6)
C3—C4—C5—C6	−0.4 (4)	C2—C1—C8—O2	175.7 (4)
C3—C4—C5—C7	178.7 (3)		

Symmetry code: (i) −*x*+1/2, −*y*+1/2, *z*.

### Hydrogen-bond geometry (Å, º)

**Table:**

*D*—H···*A*	*D*—H	H···*A*	*D*···*A*	*D*—H···*A*
C8—H8···O1	0.93	2.42	2.749 (3)	101
